# Cytokinesis in Eukaryotic Cells: The Furrow Complexity at a Glance

**DOI:** 10.3390/cells9020271

**Published:** 2020-01-22

**Authors:** Roberta Fraschini

**Affiliations:** Dipartimento di Biotecnologie e Bioscienze, Università degli Studi di Milano-Bicocca, Piazza della Scienza 2, 20126 Milano, Italy; roberta.fraschini@unimib.it; Tel.: +39-02644-83540

**Keywords:** cell division, aneuploidy, MEN network, bud neck, model organism, tetraploidy, CIN

## Abstract

The duplication cycle is the fascinating process that, starting from a cell, results in the formation of two daughter cells and it is essential for life. Cytokinesis is the final step of the cell cycle, it is a very complex phase, and is a concert of forces, remodeling, trafficking, and cell signaling. All of the steps of cell division must be properly coordinated with each other to faithfully segregate the genetic material and this task is fundamental for generating viable cells. Given the importance of this process, molecular pathways and proteins that are involved in cytokinesis are conserved from yeast to humans. In this review, we describe symmetric and asymmetric cell division in animal cell and in a model organism, budding yeast. In addition, we illustrate the surveillance mechanisms that ensure a proper cell division and discuss the connections with normal cell proliferation and organs development and with the occurrence of human diseases.

## 1. Introduction

Eukaryotic cells duplicate themselves through several rounds of a process, called mitotic cell cycle. The cell cycle is made of four phases: gap 1 (G1), DNA synthesis (S), gap 2 (G2), and mitosis (M). During mitosis, or cell division, the duplicated DNA filaments condense into chromosomes (prophase), bind the mitotic spindle in a bipolar way, and then align at the metaphase plate (metaphase); later, sister chromatids are separated and start to migrate to opposite poles of the cell (anaphase) and during telophase they conclude their movement and they decondense. After the nuclei have segregated, cytoplasm separation concludes cell division (cytokinesis). All of these events must occur in a very precise and coordinated way to preserve genetic stability, which, in turn, is essential for cell viability. Indeed, cytokinetic failure can cause multinucleate or aneuploid cells that can drive oncogenic transformation [1]. 

Given the importance of cytokinesis for cell viability, the basic processes of cytokinesis and several key proteins are evolutionarily conserved, for this reason, studies on model organisms are precious. The nematode *C. elegans*, embryos and cultured cells of the insect *D. melanogaster*, amphibian *Xenopus* eggs, and cultured mammalian cells are the mainly used animal models. The unicellular yeasts *Saccharomyces cerevisiae* and *Schizosaccharomyces pombe* are also very useful for unraveling the general principles of eukaryotic cell polarization and cytokinesis [2].

## 2. Symmetric vs. Asymmetric Cell Division

**Figure 1:**
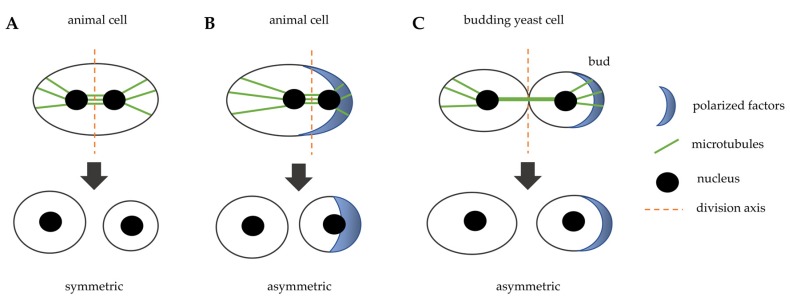
Schematic representation that illustrates symmetric and asymmetric cell division in animal cells and in budding yeast cells. (**A**) the spindle is positioned in the middle of the cell and induces a symmetric division in animal cells. (**B**) cytoplasmatic factors are positioned in a specific site of the cell, they orient the spindle that induces an asymmetric division in animal cells. (**C**) in budding yeast, polarity factors localize at a specific site of the cortex and induce bud emergence, the bud neck is the future division site and the spindle is positioned perpendicularly with respect to the division axis.

In most cases, cell division is carried out thanks to a cleavage that occurs in the middle of the mother cell, perpendicularly to the axis of chromosome segregation to allow for equal division of the genetic material. This process leads to an equal partition of the cytoplasm and it is called “symmetric cell division” (Figure 1A). After anaphase onset, the position of the spindle allows for the assembly of a cleavage furrow that specifies the division plane. This plane is defined by different types of signals, activating and inhibitory, from aster microtubules (MTs) to the cortex and from the spindle midzone to the equator. There is evidence that a negative signal is also generated at the poles for inhibiting cleavage furrow formation [3]. 

However, during embryonic development, in stem cell division in adult organisms and in some model organisms an asymmetric cell division occurs, caused by a specific mitotic spindle orientation that drives unequal cleavage. Asymmetric cell division drives cellular fate during development, indeed one daughter cell will differentiate and the other will continue to proliferate, and it is also very important for proper tissue morphogenesis. During development, asymmetric cell divisions allow for the correct tissue shape, for elongated or branched tissues and lumen in epithelial tubes. Oriented cell division contributes to accurate tissue formation, even after body development. How asymmetric cell division influences cell fate is well documented in *Drosophila* sensory organ precursor, neuroblasts, and germ line, in *C. elegans* embryos and in mammalian neuronal development, hematopoiesis, and stratification of the epidermis [4,5].

The asymmetric cell division implies the polarization of several factors inside the cell that create the asymmetry of the cytoplasm (Figure 1B). The cleavage plane is then specified by the asymmetric position of the mitotic spindle that results from the interaction of microtubules asters emanated from the centrosomes with actin and cortical proteins [6]. Rho family GTPases play an essential role in animal cell polarization. However, this process involves multiple GTPases and regulatory protein complexes, such as by Par, Crumbs, and Scribble. Polarity complexes form signaling centers that recruit Rho GTPases to specific membrane sites, and there it controls cell shape and function by regulating the actomyosin cytoskeleton and directing recycling endosome trafficking [7,8].

Even the model organism budding yeast divides asymmetrically, as the daughter cell originates from a bud that derives from the mother cell (Figure 1C). The bud neck is the place where cells will divide and it is not determined by spindle positioning, but it occurs very early during the cell cycle, at the moment of bud emergence. The rise of the bud occurs during late G1 phase of the cell cycle at a specific site of the cortex that is determined by the localization of several polarity factors. In particular, the Rho GTPase family member Cdc42 activity regulates polarized vesicle trafficking, cytoskeletal architecture remodeling, and the activation of signaling cascades, including MAPK [9]. In budding yeast, the mitotic spindle is formed after cell division site determination and before mitosis: a bipolar spindle takes shape during S phase and then it must be aligned perpendicularly with respect to the mother-bud axis to ensure proper chromosome partitioning during mitosis [10]. This asymmetric cell division is very helpful in studying the mechanisms that drive asymmetric mitosis in animal cells, since they are evolutionarily conserved. In addition, the mother cell retains markers of aging, so the yeast daughter cell can be considered to be similar to the stem cell that proliferates, whereas the mother cell gets old as the cell that stops dividing and differentiates. 

## 3. Cytokinesis in Animal Cells

In animal cells, at anaphase onset, the mitotic spindle forms a dense array of MTs, called central spindle. After chromosomes segregation, the plasma membrane starts to ingress at the cell equator and the central spindle and asters define this position, and then a cleavage furrow is formed that separates the daughter cells. The furrow consists of a contractile ring or cytokinetic ring (CR), a stable structure mostly made by actin and myosin II, a non-muscle motor protein, which is connected with the plasma membrane. At the molecular level, Rho proteins and their regulators, GAP and GEF, the protein kinases Aurora B and Polo accumulate at the equator (division plane) of the cell and signal formins recruitment that allows for the nucleation of filamentous actin (F-actin) and the localization of myosin II. Myosin II recruitment does not require its ATPase activity [11], but needs the phosphorylation of its light chain [12]. Microtubules spatially regulate the Rho pathway and kinases with molecular mechanisms that are not fully understood. 

The cleavage furrow contains also scaffolding proteins, such as anillin and septins. Anillin associates with F-actin, myosin, septins, and activated RhoA, it localizes to the furrow at early stages of cytokinesis, and it is important for its stability, but it is not essential for its ingression [13]. Instead, it might stabilize the furrow and be important for later stages of cytokinesis. Indeed, anillin remains in the cytoplasmic bridge, even after myosin and actin have dissociated. Another class of scaffolding proteins are septins: they are GTP-binding proteins that form filaments and localize to the cytokinetic ring [14]. Several human septins localize to the central spindle and midbody during anaphase and cytokinesis. Septins participate in the regulation of actin and microtubule dynamics, directly interact with anillin, and allow for the full activation of myosin that is necessary for cytokinesis. Interestingly, septins may form a barrier that restricts the diffusion of membrane proteins in the furrow since they directly bind the plasma membrane [15]. 

CR are stable biochemical structures that can be isolated and are able to contract in vitro [16], however the precise ultrastructure of actin and myosin II and the mechanism of ring contraction are unsolved problems. Recent studies took advantage of super-resolution three-dimensional (3D) structured illumination light microscopy and transmission electron microscopy (TEM) technologies to reveal CR structure of sea urchin embryos. They showed that actin within the CR is organized in antiparallel linear filaments that form an array parallel to the division plane. Myosin II filaments are concomitantly reorganized with the maturation of the ring in an actin-dependent manner. Myosin II filaments are head-to-head chains that are associated laterally with their long axis parallel to the cleavage plane [17]. These findings support a mechanism of CR contraction that involves the sliding of filaments in a purse-string model: filaments sliding shortens the ring producing a force that drives furrow ingression. 

Inward directed forces that are generated from the cytoskeleton and new membrane deposition promote furrow ingression. During this process, actin is specifically depolymerized at the equator due to actin-depolymerization factor (ADF) and cofilin [18]. Myosin II also has a rapid turnover, depending on the balance of recruitment and dissociation [19]. Actin and myosin II dynamics lead to a decrease of CR volume. CR assembly and contraction must be coordinated with membrane deposition. Indeed, plasma membrane deposition is essential for cleavage furrow formation and completion, and CR must be considered not only as force-generating machinery, but also as a landmark for vesicle delivery to the division site. 

During furrow ingression, new membrane is added locally by vesicle trafficking and both the secretory pathway and the endocytic pathway are involved. Golgi derived vesicles bring new membrane and proteins to the furrow, whereas recycling endosomes and the endocytic pathway are important for both membrane remodeling during furrow ingression and for the final steps of cytokinesis [20,21,22]. Studies in *C. elegans* and *Drosophila* revealed that the deposition of new furrow membrane requires astral microtubules and release of calcium, indicating that astral MTs not only specify positioning of the contractile ring, but also direct the delivery of new membrane to the furrow later during cytokinesis [23].

Although CR generates force to conclude cytokinesis, in some cases cells are also able to divide in the absence of a functional CR [24,25]. For example, in animal cells, the force for cell separation can derive from pulling on the substrate [26,27], since round mitotic cells maintain some substrate attachment and, in most cases, animal cells are attached to a substrate or to other cells when they divide.

Scission or abscission is the final step of cytokinesis and requires remodeling of the plasma membrane to separate sister cells. The evolutionary conserved Endosomal Sorting Complex Required for Transport (ESCRT) proteins drive this process [28]. Abscission occurs after CR has completely contracted, myosin II and filamentous actin have dissociated, and the furrow has formed an intracellular bridge rich in antiparallel microtubules with a diameter of 1 μm, the midbody. This structure is essential for proper cytokinesis completion that requires a plasma membrane remodeling that is driven by Anillin and septins, possibly fusing transport vesicles. Interestingly, a centrosome component, centriolin, has a role in vesicle targeting and fusion in the bridge [29]. Mammalian centriolin is the homologue of budding yeast Nud1 and of fission yeast Cdc11 that are MEN/SIN components that regulate mitotic exit and cytokinesis (see detailed description in Section 5). Likely, all of these proteins are involved in cytokinesis completion. In order to complete abscission, the activity of microtubule-severing proteins cuts the interdigitating MTs in the midbody, causing its breakage [30].

## 4. Cytokinesis in Budding Yeast

Cytokinesis can be studied in model organisms since several proteins and most basic cytokinesis processes are evolutionarily conserved. Table 1 shows a list of evolutionarily conserved proteins. *Saccharomyces cerevisiae* is a unicellular eukaryotic organism that offers many experimental advantages, among which: a fast cell cycle, easy and quick growth in solid and liquid medium, cellular morphology that changes during different cell cycle phases, haploid and diploid cycle, easy genetic manipulation, and analysis. An interesting feature is that *S. cerevisiae* divides asymmetrically, with the daughter cell being generated as a bud from the mother cell body. During late G1 phase of the cell cycle, an intense protein relocalization to a specific place of the cell cortex induces a strong polarization of the cells and culminates in bud emergence. 

The Rho-type GTPase Cdc42 concentrates at the site of bud emergence, together with its GEF Cdc24 and its GAP, and activates its effectors that organize the actin cytoskeleton to drive polarized growth and regulate the bud neck formation [31].

### 4.1. Bud Neck Formation

**Figure 2:**
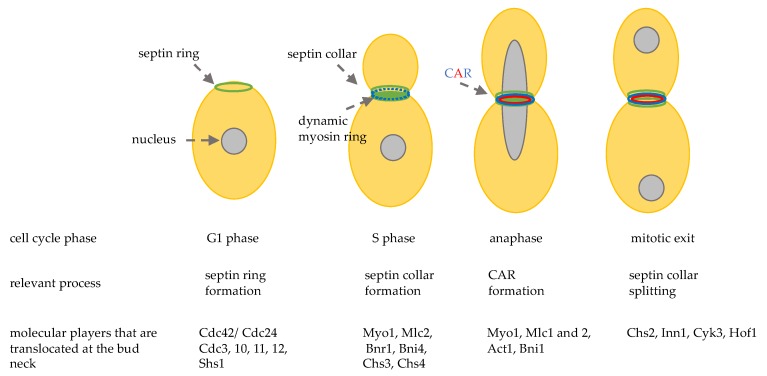
Schematic representation of the sequential localization at the division site of important cytokinesis players. CAR: contractile actomyosin ring.

The bud neck is the site that separates the mother cell from the daughter cell, it is not only a spatial point, but it has a very complex structure (Figure 2). In late G1, the first recruited proteins are septins: 5 GTP-binding proteins (Cdc3, Cdc10, Cdc11, Cdc12, and Shs1) that form linear apolar filaments that assemble into 3D structures that are highly dynamic during mitosis [32]. Septins filaments associate with the cell membrane and they form a ring around the bud neck during bud emergence and their function is essential in maintaining bud neck structure. During the progression of the cell cycle, the septin ring changes its shape becoming a rigid hourglass structure, and then splitting into two rings at the final steps of cytokinesis [33]. Septins rearrangements are regulated by protein-protein interactions and by phosphorylation/dephosphorylation and sumoylation events [34]. Septin ring acts as a platform for other proteins and a signaling landmark for actin cytoskeleton filaments that drive mitotic spindle positioning and orientation. In addition, several protein kinases that are important for cell cycle progression and cell cycle chekpoints are associated with septins and their localization at the bud neck depends on septins [35]. It is important to point out that septins also act as a cortical barrier to block movement of membrane proteins thus helping to build cellular compartmentalization [36].

During late G1 and S phase, septins recruit myosin II heavy chain, Myo1, and the light chain Mlc2, the formin Bnr1 [37], and the scaffold protein Bni4. After anaphase onset Hof1, Iqg1, Inn1, Cyk3, the regulatory chain Mlc1, and the formin Bni1 are localized at the bud neck and in late anaphase a functional actomyosin ring is formed, thanks to actin recruitment (Figure 2) [38].

### 4.2. CAR Formation and Contraction 

Budding yeast cytokinesis requires the formation and contraction of an actomyosin ring, even if the bud neck is very narrow (1 μm). The contractile actomyosin ring (CAR) is a structure composed by structural and regulatory proteins, the most important being actin and myosin. 

Actin is encoded by the essential gene *ACT1*, it is an ATP-binding protein that can form filaments that have a polarity that is built during their formation. In yeast cells, actin can form three types of structures: actin patches, actin cables, and an actin ring [31]. Actin patches are important for cell polarity formation and maintenance. Actin cables serve for intracellular transport of vesicles, organelles, and mRNA, and for mitotic spindle alignment. The actin ring is a structure that forms transiently at the mother-daughter neck, it binds myosin, and is important for cytokinesis. 

**Figure 3:**
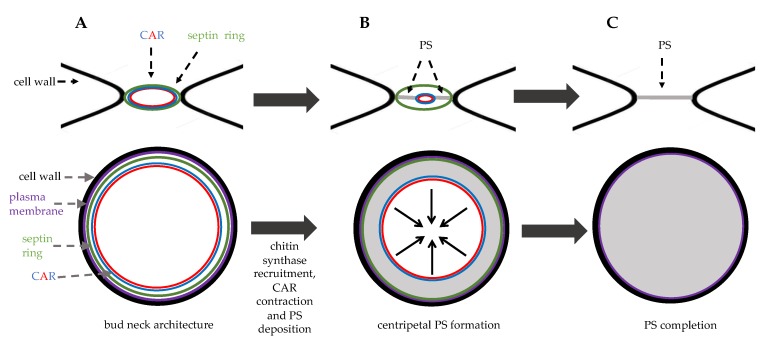
Bud neck structure during cytokinesis. Top panels: side view, bottom panels: top view. (**A**) in late anaphase septin ring and the contractile ring (CAR) are assembled at the bud neck. (**B**) in late anaphase the chitin synthase Chs2 is recruited to the bud neck and the primary septum (PS, grey area) is synthetized centripetally. (**C**) just before cell separation PS deposition is completed leading to physical dissociation of mother and daughter cytoplasms.

The second essential element of CAR is the class II myosin heavy chain, hereafter called myosin, being encoded by *MYO1* gene. Myosin is the motor that slides actin filaments and induces ring contraction [39]. The catalytic domain is located in the N-terminus, while the C-terminus contains a coiled-coiled domain that can be recruited to the site of division and it is sufficient for CAR constriction and cytokinesis. Myo1 is regulated by two light chains, an essential light chain (Mlc1) and a regulatory light chain (Mlc2). Myo1 is recruited to the emergent bud site, being present as a ring at the bud neck until cytokinesis is completed and then disassembled after contraction. In late anaphase, a complete CAR is formed around the bud neck (Figure 3A), and, after completion of previous cell cycle events, it can contract. This process does not need Myo1 motor activity and it involves a decrease in Myo1 levels and actin depolymerization regulated by cofilin, like in animal cells (see paragraph 3). 

After mitotic exit, these events concomitantly occur: CAR contracts symmetrically, membrane invaginates, and a septum is formed in a centripetal way. 

### 4.3. Cell Wall Deposition 

Since yeast cells have a cell wall, in order to complete cell separation, two single plasma membranes and a septum must be formed. Specific proteins are expressed in order to achieve this task (Table 2). Even if budding yeast division site is very thin, cell division involves membrane deposition and vesicle recruitment at the division site. In concomitance with CAR contraction, new membrane is deposited, and this ensures that the chitin septum forms correctly and efficiently. One important role of membrane deposition is to deliver integral membrane proteins that forms the septum: the chitin synthases, transmembrane proteins that polymerize chitin chain that is extruded by the plasma membrane. 

**Figure 4:**
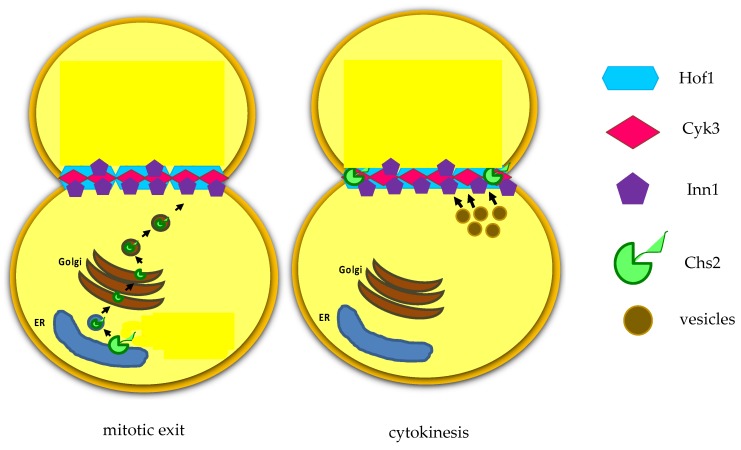
After mitotic exit, the chitin synthase Chs2 is translocated from endoplasmic reticulum (ER).

*S. cerevisiae* expresses three chitin synthases (Chs1, 2, and 3) with different roles. Chitin synthase Chs1 is a repair enzyme, while chitin synthase Chs3 is recruited to the bud neck during bud emergence and it is important for the integrity of the division site [40]. Chs3 activation requires its binding to the regulatory subunit Chs4 that physically interacts with the scaffold protein Bni4. The chitin synthase Chs2 is stored in the endoplasmic reticulum and it is delivered to the bud-neck during late anaphase in secretory vesicles [41], it is inserted in the plasma membrane and then builds the primary septum (PS), essentially a chitin disk (Figure 4) [42]. PS is deposited in a centripetal way (Figure 3B).

Recent findings indicate that Inn1 and Cyk3 regulate Chs2 catalytic domain, thus controlling chitin deposition during cytokinesis [43]. As PS is a fragile structure, after its deposition (Figure 3C), 1,3-beta-D-glucan synthases synthesize secondary septa (SS) on either side of the PS [44]. SS are glucan-rich and they have a molecular structure similar to the cell wall.

Since Chs2, Chs3, and Fks1 are localized at the division site before mitotic spindle disassembly, their activation must be tightly controlled to avoid premature spindle breakage; this task is reached by multiple regulations, among which a balance between enzyme localization and their endocytosis [45].

### 4.4. Cell Separation

**Figure 5:**
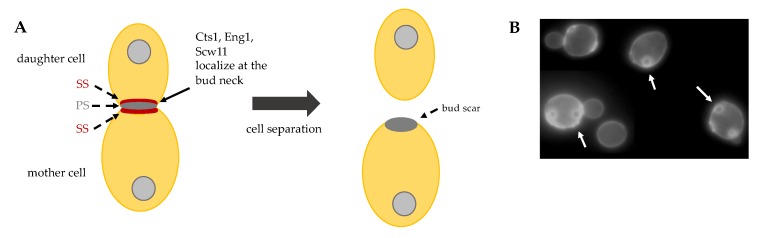
(**A**) Schematic representation of cell separation: after primary septum (PS) and secondary septa (SS) formation, Cts1, Eng1 and Scw11 activity at the daughter bud neck allows for cell separation. (**B**) Budding yeast exponentially growing cells stained with Calcofluor White. On the surface of mother cells these are several scars, chitin-containing rings (arrows), originated during cell division. New born daughter cells do not show bud scars.

The final step of cytokinesis is cell separation, the event that irreversibly divides the mother from the daughter cell. Cell separation requires the partial degradation of the septum, which occurs from the daughter cell side and leaves a sign, called bud scar, on the mother cell surface (Figure 5B). In order to degrade PS and SS partially, endochitinase Cts1 and glucanases Eng1 and Scw11 are activated specifically from the daughter side of the bud neck (Figure 5A) [46].

The activity of these hydrolytic enzymes is tightly regulated: they are specifically transcribed in the daughter cell and are correctly delivered at the daughter bud neck by the Golgi pathway. The regulatory proteins Cbk1 and Mob2 localize to the neck and to the daughter nuclei at the end of mitosis [47], in particular they form a complex with the transcriptional factor Ace2 and prevent its export from the nucleus, thus ensuring daughter-specific expression of cell separation factors [48]. In addition, Cts1 is post-translationally modified and delivered to the daughter bud neck through the Golgi pathway [49]. Recently, a new level of Cts1 regulation has been described: upon incomplete septation, Fir1 inhibits Cbk1 activity, thus blocking production and secretion of septum degrading enzymes [50]. This mechanism inhibits cell separation until septation is properly executed and, therefore, enforces cytokinesis order of events.

Cell separation is the very last event of the cell cycle and it can have great implications in cell survival. Indeed, separation of daughter from the mother must occur in correlation with other cell cycle events and a well-organized trilaminar septum structure must be formed before cell separation machinery is activated in order to obtain vital progeny. In case of overactivity of Cts1, a hole in the cell wall can be formed, in this case Chs1 is activated and repairs the cell wall at the daughter side [51].

To sum up, the successful completion of cell separation depends upon the precise construction of the trilaminar septum in coordination with the division cycle, and then on the temporal and spatial regulation of chitinase and glucanase action.

## 5. Checkpoint Pathways that Control Cytokinesis

Cytokinesis must be tightly coordinated with the nuclear cycle in order to maintain genetic stability. Eukaryotic cells have evolved several checkpoints and checkpoint-like mechanisms that preserve the integrity of cell division.

The mitotic checkpoint is an evolutionarily conserved signaling pathway that blocks mitosis progression in case of problems in the mitotic spindle, in chromosome attachment to MTs, or in presence of mistakes in chromosome segregation. In budding yeast, the division site is determined before mitotic spindle formation, which implies that the bipolar spindle must be properly positioned at the bud neck and aligned perpendicularly with respect to the division axis before anaphase onset. Two pathways direct spindle positioning, the dynein pathway and Kar9 pathway, and the spindle orientation checkpoint (SPOC) blocks mitotic exit and cytokinesis in the case of defects [52]. If the checkpoint fails, cytokinesis occurs, even if the nucleus divides into the mother cell, thus causing the formation of aneuploid cells. The target of the SPOC is Tem1, the G protein at the top of MEN pathway.

Mitotic Exit Network (MEN) in *S. cerevisiae* and Septation Initiation Network (SIN) in S. *pombe* are two signaling pathways that coordinate mitosis progression from anaphase onset to cell separation. MEN is a signaling cascade with the GTPase Tem1 at the top, several protein kinases, among which the Hippo-like kinase Cdc15 and the LATS-like kinase Dbf2, and the final target is the phosphatase Cdc14. MEN pathway leads to complete Cdc14 activation that causes a decrease in mitotic kinase activity (Cdk1), triggering mitotic exit [53]. Before anaphase onset, MEN components localize at the spindle pole bodies (the yeast centrosomes), but later they are found at the bud neck, where they ensure that mitotic exit only occurs after acto-myosin ring contraction and septum deposition [54]. In addition, in telophase, Cdc14 dephosphorylates cytoplasmic and bud-neck associated targets of Cdk1 and contributes to reorganize actin cytoskeleton and target secretion vesicles to the bud neck [55,56]. In *S. pombe* the SIN controls CAR contraction and septum deposition, rather than mitotic exit and blocks septation in the case of problems [57]. Homologs of Cdc14, MEN, and SIN components are also found in other yeasts: *Candida albicans, Aspergillus nidulans,* and *Ashbya gossypii.* Budding yeast Cdc14 is conserved in higher eukaryotes, however these homologs do not seem to control mitotic exit, rather they are involved in other processes, such as DNA replication, DNA damage repair, nuclear organization, mitotic entry, mitotic spindle assembly, and cytokinesis [58]. In *Drosophila* and human cells, MEN homologs are part of the Hippo pathway that controls centrosome duplication, cell proliferation, and apoptosis [59,60]. The conserved Hippo pathway inhibits cell proliferation by the activation of LATS1 and LATS2 kinases and by p53 stabilization (see below).

Mammalian cells have a checkpoint that arrest cells in mitosis in response to several problems in order to preserve genetic stability and cell survival, rarely cells can escape this arrest and enter mitosis without chromosome segregation, thus becoming tetraploid [61,62]. Importantly, another checkpoint control is active in human cells to recognize cytokinesis failure and induce proliferation arrest in G1. Indeed, in tetraploid cells, arising either by failure of mitotic spindle or of cytokinesis, the checkpoint induces a p53-dependent cell cycle arrest, thus preventing the proliferation of aneuploid cells and carcinogenesis [63]. However it is not clear how, at which stage and by which signal (from extra chromosomes or extra centrosomes) p53 is activated by tetraploidy. Interestingly, cytokinesis failure activates the Hippo pathway: LATS2, which is an important kinase of Hippo pathway, translocates from centrosomes to the nucleus and stabilizes p53 in the presence of additional centrosomes [64,65]. In addition, extra centrosomes can activate RAC1, which is known to antagonize RhoA, which leads to LATS2 activation [66]. If p53 function is lost, cells can override the cell cycle block that is induced by tetraploidization, accumulate chromosome aberrations, and start to proliferate without control.

Cytokinesis can be blocked even when the furrow started to ingress: the abscission checkpoint delays cytokinesis completion and can even drive the regression of the cleavage furrow in the presence of chromatin bridges or lagging chromosomes [67]. The abscission delay is dependent upon the localization of the Aurora B kinase at the midbody. Aurora B translocates from kinetochores to the division site after anaphase onset. At the midbody, activated Aurora B phosphorylates the ESCRT-III subunit Chmp4c, but the molecular mechanism that delays abscission is not completely understood.

Human cells are well equipped with several pathways that block tetraploid cells proliferation since cytokinesis failure can be tumorigenic. However tetraploidization is used by evolution and it can be important for certain tissues to gain new advantageous traits, such as resistance to drugs or stimuli (see also paragraph 6).

Interesting data suggest new connections between cytokinesis and DNA damage, in particular cytokinesis might be regulated in response to DNA damage. Damaged DNA should not be segregated, so, in this case, cytokinesis must be blocked to prevent the cleavage furrow from cutting damaged DNA. Proteins that are involved in DNA repair, such as BRCA2 and BCCIP, may be directly involved in cytokinesis, as their deficiency induces cytokinetic abnormalities [68]. The polo kinase Plk1 is an important regulator of mitosis and cytokinesis and it is also a modulator of the DNA damage checkpoint [69]. The budding yeast DNA damage checkpoint kinase Rad53 localizes at the division site and associates with septins [70]. Other experiments suggest that DNA damage pathways may regulate cytokinesis proteins by modulating their the expression or their post-translational modifications, as p53 and Rb pathways inhibit the expression of cytokinetic proteins, such as Plk1, ECT2, anillin, and survivin [71,72], and BRCT inhibits Aurora B kinase activity by Poly(ADP-ribosyl)ation in response to DNA strand breaks [73]. It is reasonable that DNA damage, and perhaps incompletely replicated DNA, activates pathways that promote DNA repair, arrests cell cycle progression, and blocks cytokinesis completion.

## 6. Cytokinesis Failure: Implication for Human Health

Cytokinesis failure leads to aneuploid cells, a typical feature of cancer cells, indeed approximately 80% of solid tumors shows chromosomal aberrations [74]. Aneuploidy refers to the gain or loss of whole chromosomes or segments of chromosomes and originates from chromosomal instability (CIN), errors in DNA repair, and problems in mitosis or cytokinesis. Aneuploidy facilitates genome plasticity that can drive the evolution of specific genotypes that promote uncontrolled cellular proliferation. In general, high CIN levels inhibit cell viability in long-term while low CIN levels allows the generation of karyotypes with a selective growth advantage [75].

Polyploidy or single chromosome abnormalities can arise from failure in mitosis or cytokinesis and lead to chromosome missegregation [76]. Accordingly, defects in these processes play an important role in tumor formation and development. Therefore, cytokinesis can be considered as a point of therapeutic intervention in cancer. Drugs that target cell division, in general, are not specific for cancer cells and this reduces their application. The same applies to drugs that block cytokinesis.

Besides cancer, aneuploidization is linked to tissue development and aging. Indeed, in brain and liver, aneuploidization seems to be part of organ development [77,78,79], supporting the concept that aneuploidy is not always detrimental, but can even be beneficial. However, the biological meaning of aneuploidization in the developing and mature brain is not clear: aneuploidy may probably promote cellular diversity in the brain, which is necessary for complex functions, such as learning and memory [78,80]. During liver development, hepatocytes become polyploid and then undergo massive chromosome loss that creates near-diploid aneuploid cells [81,82]. This process might grant the liver selective advantages against several stresses.

The connection between aneuploidy and aging is very interesting, as aging is linked to chronic diseases and declining health [83,84]. Aneuploidization in aging cells has been well documented for oocytes and it is one of the causes of female infertility [85]. In addition, mutant mice that were prone to aneuploidy developed some of age-related pathologies, such as sarcopenia, cataracts, fat loss, impaired wound healing, reduced dermal thickness [86]. Moreover, patients that suffer from mosaic variegated aneuploidy (MVA) syndrome have increased risk for childhood cancers [87], but also growth retardation, cataract, and facial dysmorphisms, probably due to tissue degeneration. Most of the MVA patients carry mutations that alter BUBR1 functionality and that cause CIN accumulation. Like aneuploidy and cancer, aneuploidy and aging is a complex interaction, with several genes contributing to CIN and aging. Several studies indicate that aneuploidization is a hallmark of aged cells [48], thus linking tissue dysfunction to age-related aneuploidy. The same study revealed that the level of aneuploidization is dependent on the proliferative index: highly proliferative tissues cells show relatively low rates and postmitotic tissues exhibit higher rates.

Therefore, it is clear that the impact of cytokinetic failure on human health is high and it is not limited to tumorigenesis, as aneuploidy is correlated to the development of neural and liver cells and age-related tissue dysfunction. Several genes that are implicated in CIN formation and propagation are involved in cancer and aging, and their dysfunction causes a variety of phenotypes that is linked to their function in chromosome segregation and cell separation. It will be interesting to study how certain types of normal tissues tolerate aneuploidy and how age-related and development aneuploidization are regulated.

## 7. Methods to Study Cytokinesis

**Figure 6:**
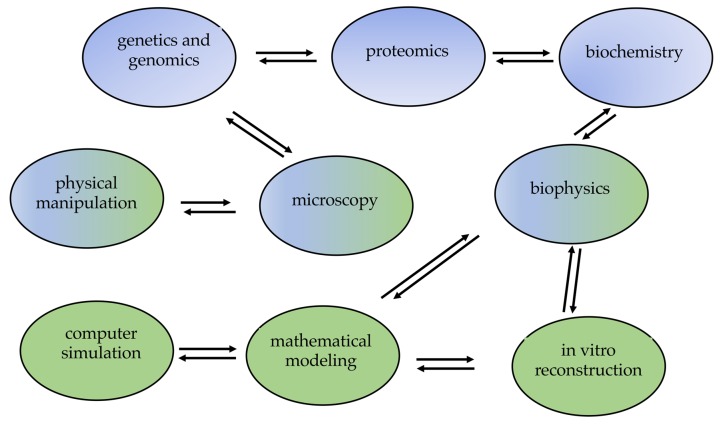
Several complementary methods can be used to identify cytokinesis proteins and to clarify cytokinesis mechanisms.

It is important to identify the molecules of the system, their interactions, and put them into pathways in order to deeply understand the complex process of cell division. To achieve these goals, several complementary approaches must be used, such as genetic and genomic approach, cell biology and microscopy, biochemistry, “-omics” technologies, and computational modeling (Figure 6).

Classic forward genetics, genetic screenings, and genetic analyses have allowed for the identification of several players in cytokinesis. In particular, unicellular eukaryotes are very useful, since they allow for the rapid analysis of the phenotypes of millions of clones of mutants carrying gene deletion or hypomorphic mutants, and this allows for establishing the correlation between proteins and specific cytokinesis steps. Conditional mutants are particularly important, since many cytokinesis genes are essential for cell viability. Temperature-sensitive mutants are extremely useful to unravel protein function, since the protein is inactivated by a fast and simple temperature shift at a specific cell cycle point.

More recently, the RNA interference (RNAi) technique has allowed for the downregulation of specific genes and development of genome-wide RNAi screens in *C. elegans*, *Drosophila* and in HeLa cell lines [88]. mRNA depletion allows for studying the functions of cytokinetic proteins in animal cells, however it lacks the time resolution that is essential to determine exactly at which step of cytokinesis a protein is required.

Proteomics is an approach complementary to genetics, which allowed for the identification of several proteins that are involved in cytokinesis that were not scored in genetic screens. However, proteomics requires the isolation of protein complexes and structures from specific cell cycle phases, which is not applicable to every cell type. In addition, it is important to obtain functional informations through other techniques since a list of proteins that are involved in cytokinesis is not very informative.

The employ of specific inhibitors is another important tool that is used to determine the role for specific proteins or structures in cytokinesis. There are drugs that can stabilize microtubules, such as taxol, or depolymerize them, like nocodazole or benomyl. These compounds can be used to block cell division. Other drugs interfere with actin, like phalloidin, which stabilize actin filaments, or latrunculin that both prevents actin polymerization and enhances its rate of depolymerization. In addition, specific Rho-kinase inhibitors can be used to impair cell polarization.

Microscopy is a great tool in cytokinesis research, classic optical and fluorescence microscopy is now assisted by new powerful digital cameras and confocal technologies. In addition, genetic modifications allow for producing strains or cell lines that express functional fluorescent proteins of different colors. These strains and cell lines can be used for time lapse analysis in living cells, for photobleaching or photoactivation experiments. Since cytokinesis is a rapid and dynamic process, these tools are extremely important to dissect cytokinetic events.

Electron microscopy and super-resolution fluorescence microscopy allowed for the analysis of CR architecture, a key player in cytokinesis. Classic electron microscopy studies have revealed the arrangement of bi-directional actin filaments along the division plane at a nanoscale level [89,90]. These filaments have also been observed by structured illumination microscopy (SIM), which, in addition, revealed Myosin II foci and their reorganization into a linear structure. Interestingly, the connection between CR and the plasma membrane has been elegantly shown by fluorescence photoactivation localization microscopy (fPALM) studies [91]. Quantitative confocal microscopy and fPALM super-resolution microscopy have been successfully used on living fission yeast cells and allowed to determine the precise stoichiometry of several proteins in the nodes, structures that concentrate at the equator of the cell before cytokinesis [92]. Hence, these techniques revealed the rearrangement of actin, myosin structures, and membrane during cytokinesis and time-lapse super resolution analyses will help to resolve the dynamics of these events in more detail. 

Biochemistry and biophysics are important for describing molecular structures, quantitative measurements, kinetics constants, and thermodynamic parameters of cytokinesis processes. The biochemistry of cytokinesis is not particularly advanced with respect to the methods described above. However, CR and some associated proteins, such as formins, have been also successfully reconstituted in vitro. Interestingly, a semi-in vitro system has been optimized to study CR contraction. The digestion of the cell wall and extraction of cytoplasmic material are used to obtain “Cell ghosts” from yeast cells by membrane permeabilization. Subsequently, in vitro assembled CR and ATP are added, and CR associates with the plasma membrane and it is able to contract. This system has been useful to define the minimal requirements for CR contraction [93].

Echinoderm zygotes, vertebrate zygotes, and cell cultures allowed for studying cytokinesis maintaining the cell environment. Physical manipulations were used to identify the signal for the position of the division plane [94] and, more recently, they are used to study CR interaction with cellular components, such as the plasma membrane, and to study the influence of several factors, such as the extracellular matrix.

In recent years, “-omics” technologies have been largely used, these include genomics, which reveals gene sequences, and transcriptomics, proteomics and metabolomics that are helpful to study the biological function of gene products. These technologies produce a huge amount of data that cannot be manually processed, so automated processes have been developed that operate on, compare, and interpret the data to create a model of pathways of interest. As a result, computerized data analyses formulate mathematical models of the biological system in study [95]. Today, several mathematical models that describe different biological processes have been developed. Mathematical models of cytokinesis help to investigate the connections among complex processes and to understand cellular response to external or internal perturbations and develop new drugs. Mathematical models are at the base of computer simulations that require less time and money than the biological experiments. However, we must keep in mind that a model is a simplification of the reality and it is only good if assumptions are valid. Accordingly, it is important to use real values for protein concentrations and association constants in order to develop models very similar to reality. Another issue to consider is the timing of cytokinetic processes (it can vary from seconds to hours) and the distance within the cell. Multiscale models must be generated in order to couple events that occur in different times and places.

## 8. Conclusions

Cytokinesis is a complex and fascinating process. In the past years, the combination of studies in different fields revealed a lot on how cells conclude their cycle and divide, thus ensuring the continuation of life. We now know dozens of factors that are involved in cytokinesis, their interactions, the meaning, and the evolutionarily conservation of pathways and proteins. However, we are far from having identified all cytokinesis players in all organisms and there is still debate about the initial steps that create protein concentration at the future division site. Similarly, it is not completely understood how the contractile ring is assembled and attached to the plasma membrane and what is the molecular signal that triggers CAR constriction and furrow formation. Accordingly, there are still a lot of unanswered questions and molecular mechanisms to unravel, which is why research on cytokinesis is still current and significant.

Humans and budding yeast are distant one-billion years of evolution, nonetheless several proteins and cellular processes are conserved. The importance of biological research in budding yeast is also highlight by three Nobel Prizes in Physiology or Medicine assigned to scientist for their discoveries of key proteins or processes in yeast. Budding yeast has been a precious model system to study cytokinesis and it is still used to discover new molecular players, functions, and molecular mechanisms. In the last years, a lot of “humanized yeasts” have been produced, which express human genes and even entire pathways [96]. These strains can be used in combination with high- throughput techniques, well developed in yeast research, to study human diseases-causing proteins, even polygenic diseases, to develop new biomarkers and design new drugs. 

## Figures and Tables

**Table 1:** Evolutionarily conserved proteins involved in cytokinesis.

Generic Name	S. *cerevisiae*	C. *elegans*	D. *melanogaster*	Humans
Structural proteins				
**Septins**	Cdc3, Cdc10, Cdc11, Cdc12, Shs1	UNC-59, UNC-61	Peanut, Sep1,2,4,5	Septin 1,4,5,6,7,8,10,11,14
**Cdc42 pathway**	Cdc42, Cdc24	CDC-42	Cdc42	Cdc42
**Actin**	Act1	ACT1, 3, 5	Actin (multiple genes)	Actin (6 genes)
**Myosin**	Myo1, Mlc1, Mlc2	NMY-2, MLC-4	Zipper, Mlc-c, Spaghetti Squash	Myosin II, Myosin ELC, Myosin RLC
**Rho proteins and regulators**	Hof1, Rho1	RHO-1, ECT-2	RhoA, Pbl	RhoA, ECT2
**Centriolin**	Nud1	CEP110	Centriolin	CNTRL (Cep110)
**Formin**	Bni1, Bnr1	CYK-1	Diaphanous	Dia1
**IQGAP**	Iqg1	PES-7	-	IQGAP1, IQGAP2, IQGAP3
**Survivin**	Bir1	BIR-1	Scapolo	Survivin
**Anillin**	Bud4	ANI-1, ANI-3	Scraps	hAnillin
Regulatory elements				
**Aurora B**	Ipl1	AIR-2	Aurora B	Aurora B
**Polo kinase**	Cdc5	PLK-1	Polo	Plk1
**MEN/SIN pathway**	Cdc14, Cdc15, Dbf2, Cbk1	CDC-14, WTS-1, YAP-1, EGL-44	Cdc14, Hippo, Mats, Warts	Cdc14A, Cdc14B, MST2, LATS1, LATS2

**Table 2:** Budding yeast proteins that are involved in the final steps of cytokinesis.

Name	Functions
**Inn1**	Couples membrane ingression with CAR contraction, regulates chitin synthase Chs2
**Cyk3**	Interacts with Hof1 and regulates chitin synthase Chs2
**Chs1**	Chitin synthase, important for septum repair
**Chs2**	Chitin synthase, mayor role in chitin primary septum synthesis
**Chs3**	Chitin synthase
**Chs4**	Regulator of chitin synthase Chs3, interacts with Bni4
**Bni4**	Required for correct septum formation
**Fks1**	Catalytic subunit of 1,3-beta-D-glucan synthase, involved in secondary septum synthesis
**Cts1**	Endochitinase, digestion of chitin in the primary septum
**Eng1**	Glucanase, digestion of the secondary septum
**Scw11**	Glucanase, digestion of the secondary septum
**Cbk1/Mob2/Ace2**	RAM signaling pathway, required for asymmetric daughter-specific transcription of chitinase and glucanases
